# Office-Based Intracordal Hyaluronate Injections Improve Quality of Life in Thoracic-Surgery-Related Unilateral Vocal Fold Paralysis

**DOI:** 10.1097/MD.0000000000001787

**Published:** 2015-10-09

**Authors:** Tuan-Jen Fang, Li-Jen Hsin, Hsiu-Feng Chung, Hui-Chen Chiang, Hsueh-Yu Li, Alice M.K. Wong, Yu-Chen Pei

**Affiliations:** From the Department of Otolaryngology, Chang Gung Memorial Hospital, Taipei (T-JF, L-JH, H-FC, H-YL); School of Medicine, Chang Gung University, Taoyuan (T-JF, L-JH, H-YL, AMKW, Y-CP); Department of Management, Graduate School, Ming Chung University (H-CC); Department of Physical Medicine and Rehabilitation, Chang Gung Memorial Hospital (AMKW, Y-CP); Healthy Aging Research Center, Chang Gung University (Y-CP); and Center of Biomedical Engineering, Chang Gung University, Taipei, Taiwan, ROC (Y-CP).

## Abstract

Thoracic-surgery-related unilateral vocal fold paralysis (UVFP) may cause severe morbidity and can cause profound functional impairment and psychosocial stress in patients with pre-existing thoracic diseases. In-office intracordal hyaluronate (HA) injections have recently been applied to improve voice and quality of life in patients with vocal incompetence, but their effect on thoracic-surgery-related UVFP remains inconclusive. We therefore conducted a prospective study to clarify the effect of early HA injection on voice and quality of life in patients with thoracic-surgery-related UVFP.

Patients with UVFP within 3 months after thoracic surgery who received office-based HA injection were recruited. Quantitative laryngeal electromyography, videolaryngostroboscopy, voice-related life quality (voice outcome survey), laboratory voice analysis, and health-related quality of life (SF-36) were evaluated at baseline, and at 1 month postinjection.

A total of 104 consecutive patients accepted office-based HA intracordal injection during the study period, 34 of whom were treated in relation to thoracic surgery and were eligible for inclusion. Voice-related life quality, voice laboratory analysis, and most generic quality of life domains were significantly improved at 1 month after in-office HA intracordal injection. No HA-related complications were reported.

Single office-based HA intracordal injection is a safe and effective treatment for thoracic-surgery-related UVFP, resulting in immediate improvements in patient quality of life, voice quality, and swallowing ability.

## Abstract 2

Supplemental Digital Content is available in the text

## INTRODUCTION

Unilateral vocal fold paralysis (UVFP) is a common complication of some surgeries, such as thyroidectomy, cervical discectomy, and esophagectomy.^1,2^ Up to 50% of cases of UVFP develop immediately after specific thoracic surgery,^3–5^ mostly associated with breathy voice, ineffective coughing, and fluid aspiration, which may in turn cause severe pneumonia and limit early oral intake. The above findings indicate the importance of early intervention to improve laryngeal function in these patients, and to alleviate the impact on their quality of life.

Acute injection laryngoplasty under general anesthesia has been used for thoracic-surgery-related UVFP.^6^ Although this was well tolerated in most patients, it failed in 1 patient as a result of a side effect of general anesthesia induction. Furthermore, patients may already be debilitated after major thoracic surgery, preventing them from undergoing conventional laryngoplasty to restore the laryngeal function.^7^ The development of digital imaging systems and distal-chip laryngoscopy has allowed the introduction of office-based injection laryngoplasty, which represents a candidate standard therapy for early phase thoracic-surgery-related UVFP. Grant et al reported that 14 of 15 patients with thoracic-surgery-induced UVFP successfully accepted awake injection laryngoplasty.^8^ They suggested that it was a helpful technique for the immediate correction of swallowing and vocal impairment. However, its use remains inconclusive because of the use of a variety of materials for injection, and a lack of objective pre- and postoperative measurements.

We recently demonstrated the benefit of office-based hyaluronate (HA) injection in acute UVFP.^9^ Voice quality and related quality of life improved immediately, with some long-term effects. However, the general health status of patients with UVFP and its causes differ, and thus its impacts may also differ. Patients with UVFP as a result of thoracic surgery are likely to have poorer health and stay longer in hospital for intensive care^10^ than those with UVFP from other causes. Some patients may be at increased risk of other complications, especially in relation to general anesthesia. The need for early treatment of UVFP and its effects should thus be evaluated for each specific cause.^9^

The effect of injection laryngoplasty may differ according to the injectable material used. The safety and effectiveness of injection laryngoplasty using HA has been reported for the management of UVFP. HA injections immediately improved the voice in patients with acute UVFP,^11^ and office-based injections have thus increased in popularity. This case-series study aimed to evaluate the feasibility of early intracordal HA injection in patients with thoracic-surgery-related UVFP and to determine the short-term postoperative outcomes. We hypothesized that HA injection for these patients can improve their voice and quality of life.

## MATERIALS AND METHODS

### Patients

**FIGURE 1:**
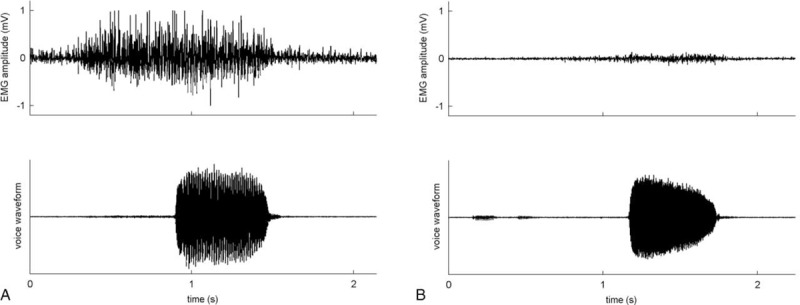
LEMG activity when an example patient is making an /eee/ with maximal effort. Upper and lower traces are LEMG and voice waveforms, respectively. (A) Activities recorded in a healthy TA-LCA muscle activity showed a normal interference pattern. (B) Activities recorded in a lesioned TA-LCA muscle activity showed a decreased interference pattern. LEMG = laryngeal electromyography, TA-LCA = thyroarytenoid-lateral cricoarytenoid.

This study was approved by the Ethics Committee and the Institutional Review Board of Chang Gung Medical Foundation, Taiwan. The study cases were selected retrospectively from a prospective patient cohort collected between August 2011 and December 2014, comprising patients with acute UVFP manifested with dysphonia, who had undergone office-based injection laryngoplasty with HA (Restylane^®^, Q-Med AB, Uppsala, Sweden) and received the follow-up assessment. The inclusion of eligibility for all patients within a period of time was used to avoid selection bias. Written informed consent was obtained from each participant before recruitment. We recruited patients with thoracic-surgery-related UVFP and received office-based HA injection. The date of thoracic surgery should be within 3 months before our first outpatient assessment. The patient's voice should be adequate before the thoracic surgery and UVFP-associated symptoms occurred within 1 month following the thoracic surgery. The diagnosis of UVFP was confirmed by both laryngoscopy and laryngeal electromyography (LEMG) (Figure 1). Exclusion criteria were diagnosis of vocal fold palsy before surgery, other etiologies that can account for their UVFP, no evidence of denervation changes in the thyroarytenoid muscle in the LEMG study, and patients unable to cooperate with evaluations.

### Assessments

At each assessment, each patient underwent quantitative LEMG (QLEMG), videolaryngostroboscopy, Mandarin Chinese-version Voice Outcome Survey (VOS) (Table 1), laboratory voice analysis, and SF-36 health-related quality of life. The protocols for LEMG and QLEMG have been described previously.^12,13^ Patients were assessed before and 1 month after HA injection.

**TABLE 1:**
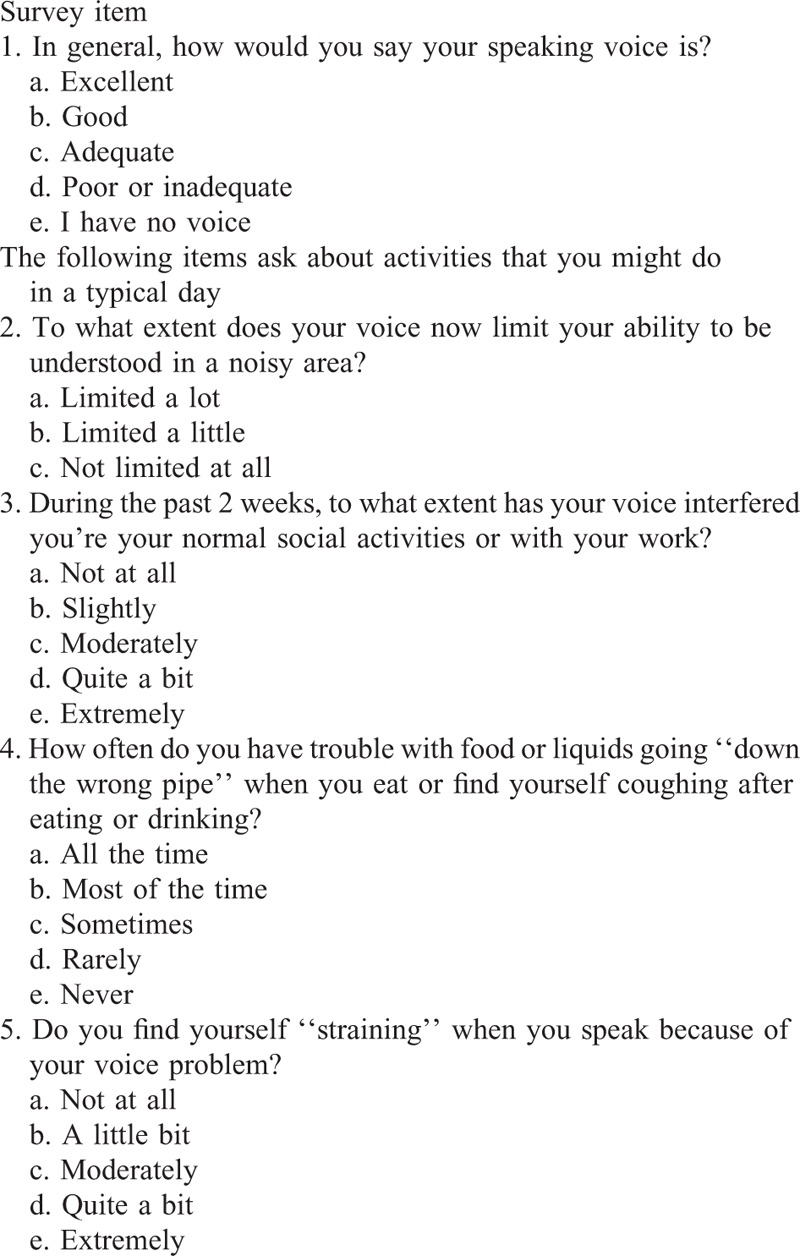
Voice Outcome Survey

### Laryngeal Configuration: Normalized Glottal Gap Area (NGGA)

The normalized glottal gap area (NGGA) was analyzed using Image J software (Image J 1.44p, National Institutes of Health, Bethesda, MA), following the method defined by Omori et al^14^ Specifically, videolaryngoscopy captured images whereas the patient vocalized /eee/ at modal pitch and regular loudness. The glottal gap was calculated (in square pixels) and membranous vocal-fold length was measured from the point of the anterior commissure to the point of the tip of the vocal process (in linear pixels). The NGGA was computed by equation (1): 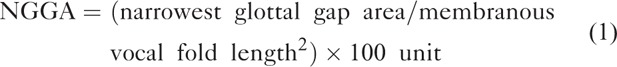


Glottal gaps were measured individually during the open and closed phases in the phonatory cycle.

### Voice Outcome Survey (VOS)

The VOS questionnaire, developed by Gliklich et al,^15^ consists 5 items that evaluate the UVFP-relative physical and social problems. Each survey item and the total scores were normalized on a 0 (worst) to 100 (best) scale. The Mandarin Chinese version of VOS questionnaire has been validated by the present research team.^16^

### Laboratory Voice Analysis

Each patient was asked to read a standard passage and a sustained vowel at a conversational pitch and loudness in a sound-insulated room. Their voice was captured using a unidirectional dynamic microphone (Shure SM48; Shure Brothers Inc, Agua Prieta, Mexico) with a distance of 10 cm between mouth and the microphone and an off-axis angle of 45°. Voice was then recorded and sampled using voice-analysis software (Computerized Speech Lab model 4300B, version 5.05; Kay Elemetrics Corp., Lincoln Park, NJ) with a sampling rate of 25.6 kHz and a 16-bit quantization. Modal fundamental frequency, perturbation of frequency (jitter) and amplitude (shimmer), and harmonic-to-noise ratio (HNR) were tabulated from the recorded voice. Each parameter reflects a specific voice dimension. The values of jitter and shimmer reflect the deviation from voice periodicity and tend to increase in patients with voice problems. HNR quantifies the amount of additive noise produced by turbulent glottal airflow and was suggested to be more analogous to the perception evaluation. The maximal phonation time (MPT) represented the longest duration of sustaining a vowel /a/. The SZ ratio was the ratio of the voicing duration of /s/ to /z/, which represents the patient's vocal control, with the ideal reference value being close to 1.0. Patients with UVFP tend to have shorter MPT and longer SZ ratio as compared to healthy subjects.

### Intracordal HA Injection (see Video, Supplemental Video, http://links.lww.com/MD/A454, Which Demonstrates Injection of the Larynx)

**FIGURE 2:**
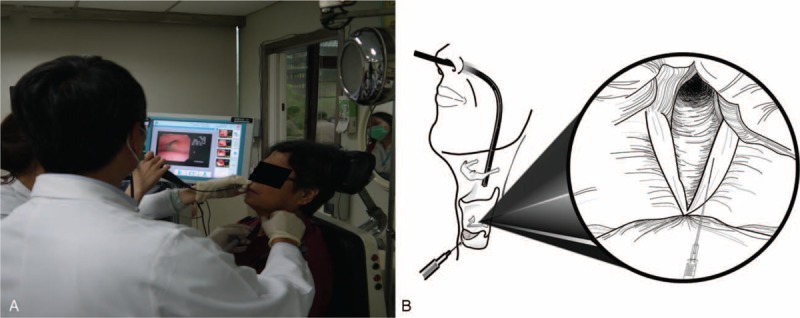
Office-based intracordal hyaluronate injection. (A) Needle positioning in right vocal cord paralysis. (B) Injection performed in the laryngology outpatient clinic. Please see supplementary information for the video clip, http://links.lww.com/MD/A454.

Intracordal HA injection is getting popular in recent years, but this procedure is an off-label use. Patients received intracordal HA injection in an office-based setting (Figure 2). Before injection, the nasal mucosa was anesthetized by spraying with 2% lidocaine with epinephrine 1:100,000, and the oral cavity and oropharynx were anesthetized by spraying with 10% lidocaine. Patients were seated upright with their neck extended. After the subject felt numbing over their throat, an assistant physician inserted a distal-chip laryngoscope (ENF V2; platform: EVIS Exera II; Olympus Optical Co, Ltd, Tokyo, Japan) transnasally to allow visualization of the glottis on the monitor. Lidocaine (0.5 mL, 2%) was injected into the subcutaneous tissue at the midpoint of the cricothyroid membrane. The needle containing HA was first placed on the para-midline point at the level of the cricothyroid junction. After the needle was transmitted through the cricothyroid membrane, it could be moved gently submucosally to confirm the location of the tip. The location of the needle was confirmed by carefully moving it forward and backward a short distance. It was essential to avoid placement of the needle tip in the airway or Reinke's space of the vocal fold. After the location had been confirmed, up to 1 mL of HA was pushed slowly into the vocal fold. The patient was asked to project their voice during and at the end of injection, to confirm the vocal fold position and satisfactory voice.

### Statistical Analysis

Data were analyzed using SPSS (PASW statistics 18, IBM SPSS Inc, IL). One out of 34 patients had missing data in laboratory voice analysis and the patient was not analyzed specifically for the laboratory voice parameters. Parameter changes over time were compared using Student's *t* tests for parametric data and χ^2^ tests for categorical data. The α-value was defined as 0.05.

## RESULTS

During the study period, 123 patients with UVFP accepted in-office HA injection postinjection assessment, among whom 20 were excluded (19 patients for missing data and 1 for normal LEMG). Among the remaining 103 patients, 34 patients were related to thoracic surgery. Patient demographics, type of surgery, and peak turn frequencies of LEMG of thyroarytenoid–lateral cricoarytenoid muscles are listed in Table 2. There were 3 times more men than women, and most of the patients suffered from left palsy, whereas only 12% had right-sided injury.

**TABLE 2:**
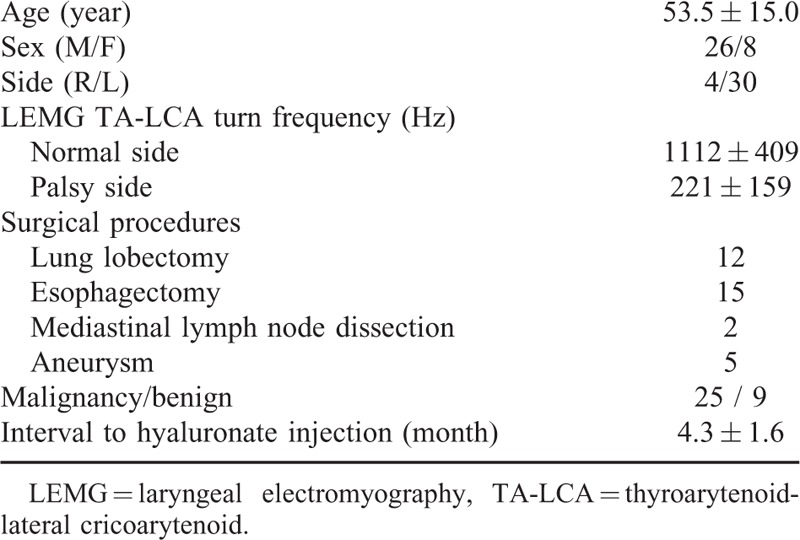
Patient Demographics

In 9 of the 34 cases, UVFP derived from surgery for benign diseases. Twenty-five patients developed UVFP after cancer surgery; 8 for lung cancer, 16 for esophageal cancer, and 1 for mediastinal malignancy. The detailed characteristics of patients in terms of cancer-related surgeries are shown in Table 3. Most cancer-related UVFPs were left-sided, but 4 were right-sided. Right vocal paralysis was associated with right upper lobectomy in 2 cases, whereas 2 others underwent mediastinal lymph node dissection to remove metastatic lymph nodes from esophageal cancer, close to the right recurrent laryngeal nerves.

**TABLE 3:**
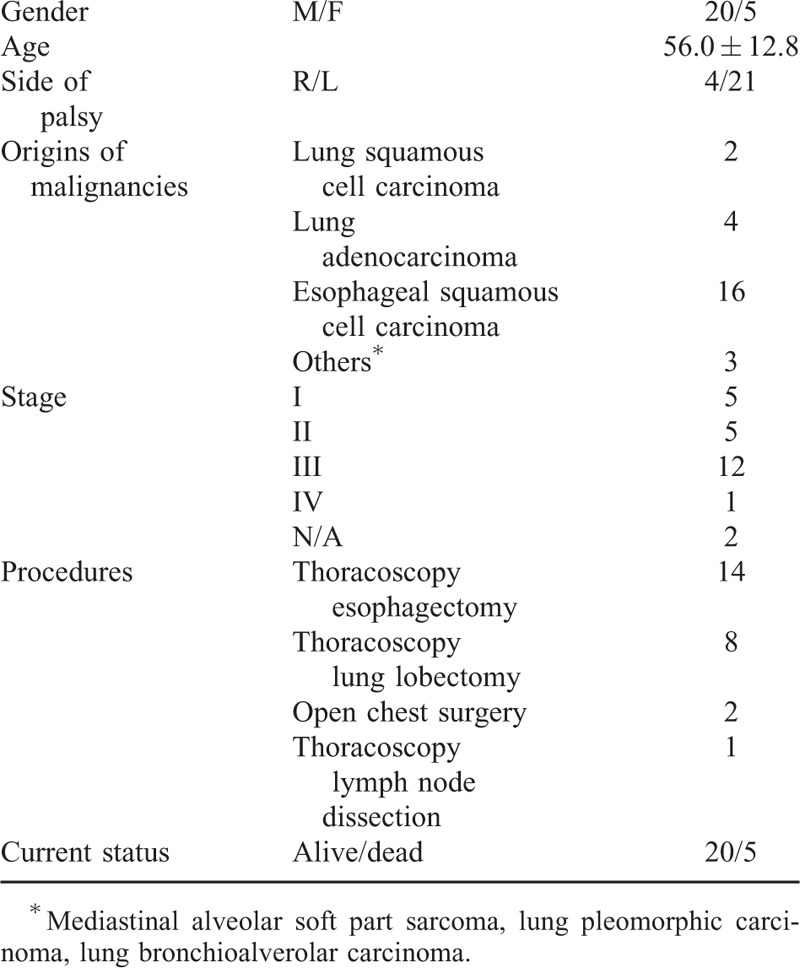
Characteristics of Cancer-Related Subjects

**FIGURE 3:**
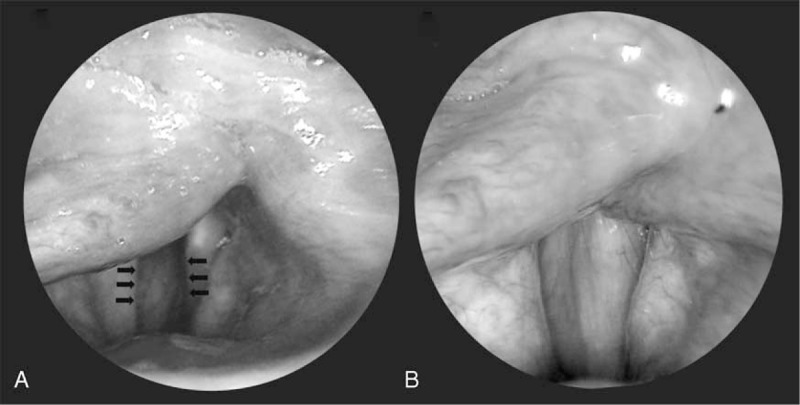
Glottal gap before (A) and 1 month after HA injection (B) HA = hyaluronate.

**FIGURE 4:**
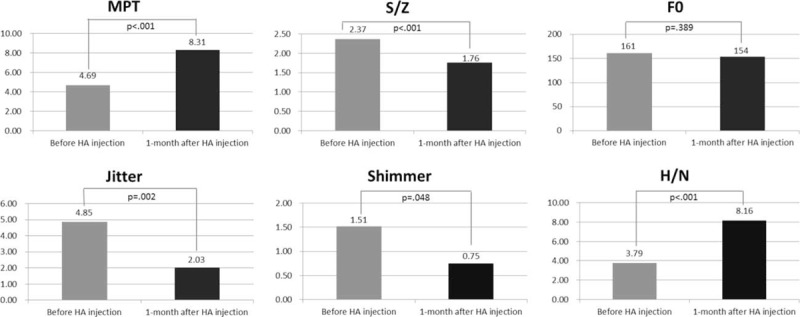
Laboratory voice analysis before and 1 month after HA injection. HA = hyaluronate, H/N = harmonic to noise ratio, MPT = maximal phonation time. ^∗^*P* < 0.05.

All patients tolerated transcervical intracordal injection of HA. The procedure lasted 12 to 20 min. There were no immediate complications, but 2 patients complained of neck ecchymosis. The glottal gaps were approximated immediately after HA injection (Figure 3). The open- and closed-phase NGGAs were improved at 1 month follow-up compared with preoperative values, from 20.87 ± 8.86 to 13.82 ± 6.65, and 9.46 ± 8.26 to 3.87 ± 3.90, respectively (*P* < 0.001). The acoustic and aerodynamic parameters measured in the voice laboratory showed significant improvements in all but the fundamental frequencies (Figure 4).

**FIGURE 5:**
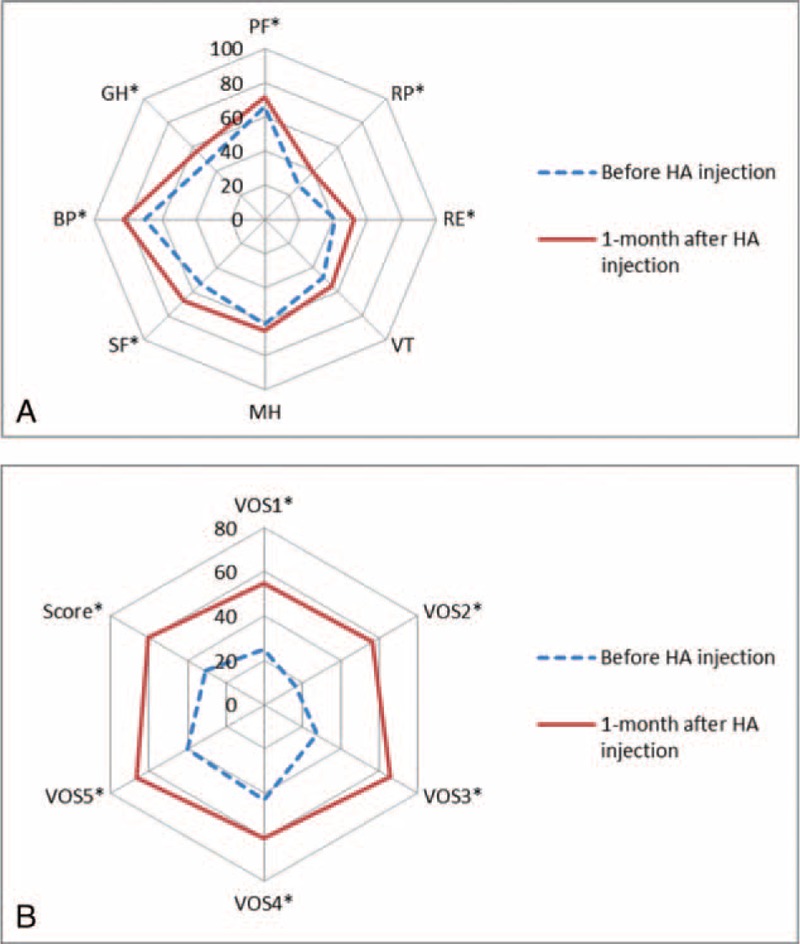
Quality of life measurements before and 1 month after HA injection. A. SF-36. B. VOS. BP = body pain, GH = general health, HA = hyaluronate, MH = emotional well-being, PF = physical functioning, RE = role limitations due to emotional problems, RP = role limitations due to physical health, SF = social functioning, VT = vitality, ^∗^*P* < 0.05. ^∗^*P* < 0.05.

Quality of life measured by SF-36 demonstrated significant improvements in the domains of physical functioning (*P* = 0.0420), role limitations owing to physical health (*P* = 0.026), role limitations owing to emotional problems (*P* = 0.017), general health bodily pain (*P* = 0.031), and social functioning (*P* = 0.001) (Figure 5A). Emotional well-being (*P* = 0.070) and vitality (*P* = 0.202) were also improved, but the differences were not significant. Voice-related quality of life measured by VOS showed significant improvements in all items (*P* < 0.001 for items 1 to 3, *P* = 0.009 for item 4, and *P* = 0.001 for item 5) and in the composite score (*P* < 0.001) (Figure 5B).

All patients were followed-up for at least 6 months, or until death. Three patients subsequently accepted permanent laryngoplasty. Five patients died during follow-up periods of 5.3 to 31.2 months (mean 13.0 ± 9.2 months).

## DISCUSSION

Recurrent laryngeal nerve injury that manifests with UVFP is a common complication of thoracic surgery. Patients with thoracic-surgery-related UVFP tend to suffer from breathy voice and swallowing disturbances, and are at increased risk of aspiration pneumonia and death. Most patients require intensive care immediately following thoracic surgery, and compromised cardiopulmonary function may make them ineligible for injection laryngoplasty under general anesthesia. Given that oral feeding may subsequently be delayed in these patients solely as a result of a correctable UVFP, office-based injection laryngoplasty represents a unique opportunity for providing a safe and effective early intervention.

Injection laryngoplasty has been an established treatment for UVFP for decades and has recently been suggested for early-phase UVFP. However, although early injection laryngoplasty with a temporary agent under general anesthesia showed promising results in patients with thoracic surgery-related UVFP, 1 of 20 cases was complicated by pneumonitis caused by induction anesthesia.^6^ Office-based injection laryngoplasty, in which the procedure is performed in awake patients, might thus offer a safe alternative method for early intervention. Indeed, we previously reported the use of office-based HA injection for acute UVFP.^9,13^ The injected HA would remain in the vocal cord temporarily^17^ and may thus not interfere with the spontaneous recovery of vocal-fold motion. Given that general anesthesia may not be tolerated by most patients immediately after thoracic surgery, office-based HA injection may help to restore their voice function, with minimal risk. Subjects demonstrated fast recovery after office-based HA injection and were able to talk and eat on the same day. The shortened recovery period also enabled patients to undergo appropriate early adjuvant therapy (eg chemoradiation therapy) for the superimposed thoracic disease.

Five patients in the current series had died by the end of follow-up, at a mean of 13 months post-HA injection, all as a result of their thoracic malignancies. Although the survival of patients with thoracic malignancies may be relatively short, the procedure nevertheless improved their life quality without the need for a prolonged hospital stay.

The present study showed that office-based intracordal HA injection was safe and effective during the early phase of UVFP and improved voice and quality of life in patients after thoracic surgery. The procedure can be accomplished within 30 min. In our experience, the main difficulty associated with awake injection is handling acid reflux and gag reflex, especially in patients after esophagectomy. Some patients are required to clear their throat several times to expose the larynx, to avoid its being obscured by secretions. We suggest performing the injection in a sitting, rather than a supine position. The patient should be instructed not to take food for at least 2 h before the procedure and should be prepared by adequate mucosal numbing.

Voice may be restored immediately following office-based HA injection. Although HA may not alter motor unit recruitment, it can correct the glottal gap, voice, and the associated quality of life immediately after injection and at 1-month follow-up. The complication of fluid aspiration may also be reduced, because the protective effect of vocal closure is re-established. Quality of life analysis also showed an immediate positive effect of early HA injection not only on physical function, but also on psychosocial well-being.

Although the injected HA is absorbed over time, it may still have a long-lasting effect if given during the early phase of UVFP, especially in patients with a large glottal gap.^18^ Friedman et al showed that 63% of patients who received HA injection within 6 months of symptom onset retained adequate voice at long-term follow-up.^19^ In addition, early HA injection reduced the need for transcervical medialization laryngoplasty in patients with UVFP.^19,20^ However, the present study focused on the short-term effects of office-based HA injection for thoracic-surgery-related UVFP, and further studies are needed to investigate its long-term outcome in these patients.

The left recurrent laryngeal nerve has a longer passage in the thoracic cage than the right recurrent laryngeal nerve, which explains why most cases of thoracic-surgery-related UVFP occurred on the left side. However, the 4 right UVFP cases in the present study resulted from thoracoscopic surgical procedures involving the upper mediastinum or right upper lobe. Popularization of the technique of minimally invasive endoscopy might be associated with an increase in the incidence of right-side vocal paralysis. It is therefore important to note the surgical anatomy of the right recurrent laryngeal nerve in cases requiring upper thoracic procedures.

## CONCLUSIONS

Office-based intracordal HA injection can provide immediate improvements in voice and swallowing in patients with UVFP, allowing an earlier return to normal social functioning. The procedure is safe and effective, and is thus highly recommended as an early intervention in these patients, including those with thoracic-surgery-related UVFP.

## References

[1] Ko HC Lee LA Li HY Etiologic features in patients with unilateral vocal fold paralysis in Taiwan. *Chang Gung MJ* 2009; 32:290–296. 19527608

[2] Pei YC Fang TJ Li HY Cricothyroid muscle dysfunction impairs vocal fold vibration in unilateral vocal fold paralysis. *Laryngoscope* 2014; 124:201–206. 23712513 10.1002/lary.24229

[3] Dimarakis I Protopapas AD Vocal cord palsy as a complication of adult cardiac surgery: surgical correlations and analysis. *Eur J Cardiothorac Surg* 2004; 26:773–775. 15450571 10.1016/j.ejcts.2004.06.003

[4] Pham V Connelly D Wei JL Vocal cord paralysis and Dysphagia after aortic arch reconstruction and Norwood procedure. *Otolaryngol Head Neck Surg* 2014; 150:827–833. 24515967 10.1177/0194599814522413 PMC4262533

[5] Pertl L Zacherl J Mancusi G High risk of unilateral recurrent laryngeal nerve paralysis after esophagectomy using cervical anastomosis. *Eur Arch Oto Rhinol Laryngol* 2011; 268:1605–1610. 10.1007/s00405-011-1679-7 21706158

[6] Graboyes EM Bradley JP Meyers BF Efficacy and safety of acute injection laryngoplasty for vocal cord paralysis following thoracic surgery. *Laryngoscope* 2011; 121:2406–2410. 21994176 10.1002/lary.22178

[7] Wright CD Zeitels SM Recurrent laryngeal nerve injuries after esophagectomy. *Thoracic Surg Clin* 2006; 16:23–33.v. 10.1016/j.thorsurg.2006.01.006 16696280

[8] Grant JR Hartemink DA Patel N Acute and subacute awake injection laryngoplasty for thoracic surgery patients. *J Voice* 2008; 22:245–250. 17067779 10.1016/j.jvoice.2006.09.001

[9] Pei YC Fang TJ Hsin LJ Early hyaluronate injection improves quality of life but not neural recovery in unilateral vocal fold paralysis: an open-label randomized controlled study. *Restor Neurol Neurosci* 2015; 33:121–130. 25588457 10.3233/RNN-140439

[10] Lodewyks CL White CW Bay G Vocal cord paralysis after thoracic aortic surgery: incidence and impact on clinical outcomes. *Ann Thorac Surg* 2015; 100:54–58. 25886811 10.1016/j.athoracsur.2015.02.021

[11] Song PC Sung CK Franco RA Jr Voice outcomes after endoscopic injection laryngoplasty with hyaluronic acid stabilized gel. *Laryngoscope* 2010; 120 (Supp l 4):S199. 21225797 10.1002/lary.21666

[12] Pei YC Fang TJ Li HY Cricothyroid muscle dysfunction impairs vocal fold vibration in unilateral vocal fold paralysis. *Laryngoscope* 2014; 124:201–206. 23712513 10.1002/lary.24229

[13] Fang TJ Pei YC Li HY Glottal gap as an early predictor for permanent laryngoplasty in unilateral vocal fold paralysis. *Laryngoscope* 2014; 124:2125–2130. 24668456 10.1002/lary.24689

[14] Omori K Kacker A Slavit DH Quantitative videostroboscopic measurement of glottal gap and vocal function: an analysis of thyroplasty type I. *Ann Otol Rhinol Laryngol* 1996; 105:280–285. 8604889 10.1177/000348949610500407

[15] Gliklich RE Glovsky RM Montgomery WW Validation of a voice outcome survey for unilateral vocal cord paralysis. *Otolaryngol Head Neck Surg* 1999; 120:153–158. 9949345 10.1016/S0194-5998(99)70399-2

[16] Fang TJ Li HY Gliklich RE Assessment of Chinese-version voice outcome survey in patients with unilateral vocal cord paralysis. *Otolaryngol Head Neck Surg* 2007; 136:752–756. 17478210 10.1016/j.otohns.2006.11.048

[17] Lau DP Lee GA Wong SM Injection laryngoplasty with hyaluronic acid for unilateral vocal cord paralysis. Randomized controlled trial comparing two different particle sizes. *J Voice* 2010; 24:113–118. 19535219 10.1016/j.jvoice.2008.05.007

[18] Fang TJ Pei YC Li HY Glottal gap as an early predictor for permanent laryngoplasty in unilateral vocal fold paralysis. *Laryngoscope* 2014; 124:2125–2130. 24668456 10.1002/lary.24689

[19] Friedman AD Burns JA Heaton JT Early versus late injection medialization for unilateral vocal cord paralysis. *Laryngoscope* 2010; 120:2042–2046. 20824787 10.1002/lary.21097

[20] Prendes BL Yung KC Likhterov I Long-term effects of injection laryngoplasty with a temporary agent on voice quality and vocal fold position. *Laryngoscope* 2012; 122:2227–2233. 22865287 10.1002/lary.23473

